# Dentate Gyrus Peroxiredoxin 6 Levels Discriminate Aged Unimpaired From Impaired Rats in a Spatial Memory Task

**DOI:** 10.3389/fnagi.2019.00198

**Published:** 2019-07-31

**Authors:** Jana Lubec, Roman Smidak, Jovana Malikovic, Daniel Daba Feyissa, Volker Korz, Harald Höger, Gert Lubec

**Affiliations:** ^1^Department of Neuroproteomics, Paracelsus Private Medical University, Salzburg, Austria; ^2^Department of Pharmaceutical Chemistry, University of Vienna, Vienna, Austria; ^3^Core Unit of Biomedical Research, Division of Laboratory Animal Science and Genetics, Medical University of Vienna, Himberg, Austria

**Keywords:** peroxiredoxin, PRDX6, aging, hole-board, proteomics, spatial memory, hippocampus, dentate gyrus

## Abstract

Similar to humans, the normal aged rat population is not homogeneous in terms of cognitive function. Two distinct subpopulations of aged Sprague–Dawley rats can be identified on the basis of spatial memory performance in the hole-board paradigm. It was the aim of the study to reveal protein changes relevant to aging and spatial memory performance. Aged impaired (AI) and unimpaired (AU) male rats, 22–24 months old were selected from a large cohort of 160 animals; young animals served as control. Enriched synaptosomal fractions from dentate gyrus from behaviorally characterized old animals were used for isobaric tags labeling based quantitative proteomic analysis. As differences in peroxiredoxin 6 (PRDX6) levels were a pronounced finding, PRDX6 levels were also quantified by immunoblotting. AI showed impaired spatial memory abilities while AU performed comparably to young animals. Our study demonstrates substantial quantitative alteration of proteins involved in energy metabolism, inflammation and synaptic plasticity during aging. Moreover, we identified protein changes specifically coupled to memory performance of aged rats. PRDX6 levels clearly differentiated AI from AU and levels in AU were comparable to those of young animals. In addition, it was observed that stochasticity in protein levels increased with age and discriminate between AI and AU groups. Moreover, there was a significantly higher variability of protein levels in AI. PRDX6 is a member of the PRDX family and well-defined as a cystein-1 PRDX that reduces and detoxifies hydroxyperoxides. It is well-known and documented that the aging brain shows increased active oxygen species but so far no study proposed a potential target with antioxidant activity that would discriminate between impaired and unimpaired memory performers. Current data, representing so far the largest proteomics data set in aging dentate gyrus (DG), provide the first evidence for a probable role of PRDX6 in memory performance.

## Introduction

Cognitive abilities such as memory may see a decline in older adults, however, there is a notable heterogeneity in cognitive functions across aged subjects. Any aged rat population can be distinguished into two subpopulations: impaired (AI) and unimpaired (AU) in spatial learning and memory tasks (Brouillette and Quirion, 2008; Menard and Quirion, 2012; Farso et al., 2013).

Aging is characterized by a progressive accumulation of oxidative damage to lipids, DNA, and protein in a wide variety of tissues (Bokov et al., 2004). According to the free radical theory of aging, mitochondria initiate most of the deleterious changes in aging. Mitochondria are not only the main producers of energy in the cells but also the principal source of intracellular reactive oxygen species (ROS). Thus, mitochondrial dysfunction and reduced expression and/or activity of naturally occurring antioxidants seems to be critically involved in both, normal and pathological aging, as well as neurodegenerative disorders.

Peroxiredoxin 6 (PRDX6) is a 1-cys member of the PRDX family that exhibits the unique combination of glutathione peroxidase and phospholipase A2 activities (Fisher, 2011). The ability of PRDX6 to reduce peroxidized cell membrane phospholipids and to replace the oxidized fatty acyl group through its phospholipase activity (Manevich et al., 2007; Rahaman et al., 2012; Fisher et al., 2016) provides a meaningful system for the repair of peroxidized cell membranes (Fisher, 2017). Several studies demonstrated the importance of PRDX6 in maintaining redox homeostasis. Loss of PRDX6 makes cells highly susceptible to stressor-induced death and causes etiopathogenesis in different tissues, while introduction of PRDX6 overrides this process (Eismann et al., 2009; Shim and Kim, 2013; Pacifici et al., 2014). Moreover, dysregulation of PRDX6 in Alzheimer (Power et al., 2008) and Parkinson’s disease has been documented (Power et al., 2002).

Most convincing evidence for a role of PRDX6 in oxidative stress in neurons comes from a report by Singh et al. (2016). The authors have shown that the expression of PRDX6 was decreased in neuronal cells under stress and cells subsequently underwent apoptosis. Using PRDX6 fused to the TAT transduction domain, they have shown that the enzyme was internalized in brain cortical neurons and this conferred resistance against oxidative stress. Neuroprotection by PRDX6 has been documented under several pathophysiological conditions as cerebral ischemia/reperfusion (Yu et al., 2013; Shanshan et al., 2017), stroke (Jia et al., 2017), hypoxia (Tulsawani et al., 2010), tau toxicity and amyloid-β-induced apoptosis of PC12 cells (Yata et al., 2011; Kim et al., 2013) and probably in CNS trauma (Buonora et al., 2015; Zhang et al., 2015). And indeed, it is text book knowledge that age-related cognitive decline can be linked to oxidative stress (Blalock et al., 2003; Fraser et al., 2005; Foster, 2006; Dröge and Schipper, 2007; Craft et al., 2012; Perluigi et al., 2014) although information on PRDX6 is limited.

The aim of this study was to elucidate the mechanisms behind “successful” cognitive aging using a large-scale quantitative proteomics approach. There is so far no published information on PRDX6 in the aging rat with experimentally proven cognitive decline. In contrast to the majority of previous publications old rats were subdivided into impaired and unimpaired groups based on performance in a spatial memory task. The results of our study revealed significantly changed PRDX6 protein levels between the two aged groups with the mean value obtained for unimpaired rats being comparable to young animals.

## Materials and Methods

### Animals

Animals were bred and maintained in the Core Unit of Biomedical Research, Division of Laboratory Animal Science and Genetics, Medical University of Vienna. The animals lived in a separate experimental room 1 week before and throughout the experiment. Rats were housed individually in standard Makrolon cages filled with autoclaved woodchips (temperature: 22 ± 2°C; humidity: 55 ± 5%; 12 h artificial light/12 h dark cycle: light on at 7:00 am). The study was carried out in accordance with the recommendations of the European Communities Council Directive of 24 November 1986 (86/609/EEC) evaluated by the ethics committee of the Medical University of Vienna, Austria. The protocol was approved by the Federal Ministry of Education, Science and Culture, Austria.

### Evaluation of Spatial Memory in the Hole-Board Test

**Figure 1:**
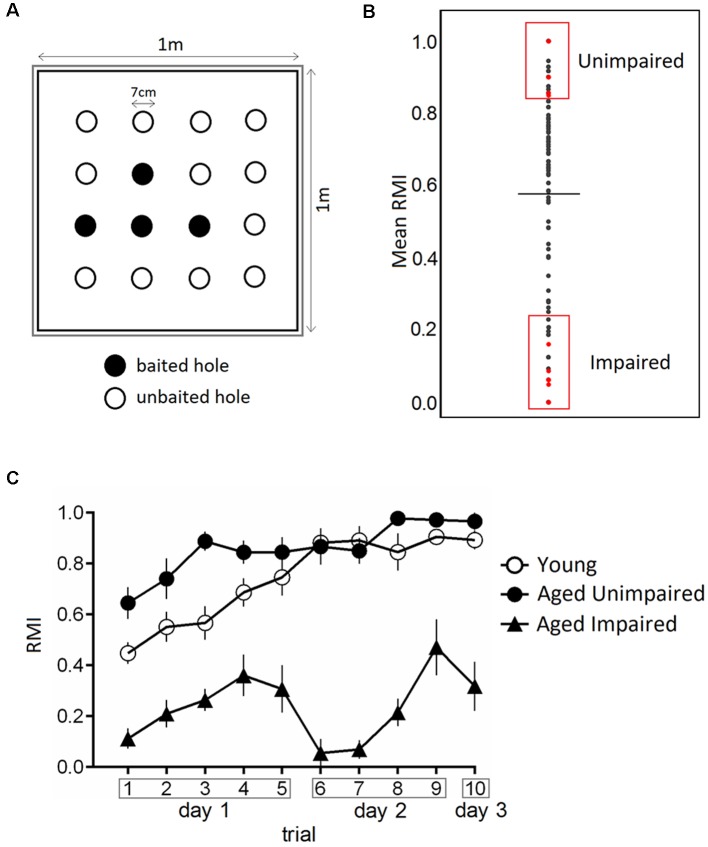
Performance in the hole-board test. **(A)** Schematic of the hole-board maze, a non-sophisticated paradigm for the evaluation of spatial memory. Animals have to learn and remember position of four baited holes during 3 days in 10 trials. **(B)** From a large cohort of 160 male Sprague–Dawley rats, 22–24 months old, impaired and unimpaired performers were selected based on their mean Reference Memory Index (RMI) derived from trial 6 and 10 ± 1 standard deviation (SD). The dashed line indicates the mean of mean RMIs. **(C)** Performance of young, aged impaired (AI) and aged unimpaired (AU) rats in the hole-board test. The data are presented as mean ± SEM, *n* = 9. AU animals performed the task comparable to young rats.

A hole-board test was performed as previously described with minor modifications (Smidak et al., 2017). The hole-board with the dimension of 1 m × 1 m was manufactured of a black plastic board bounded by a translucent plexiglass wall, with proximal spatial cues (black/white symbols) and surrounded by room structures which served as distal cues. Four out of 16 regularly arranged holes (diameter and depth 7 cm) were baited (dustless precision pellets, 45 mg, Bioserv^®^, Flemington, NJ, USA). The pattern of baited holes remained the same during the entire test (Figure 1A). A second board below the first was scattered with food pellets to avoid olfactory orientation. Ten minutes handling sessions per day for 4 days prior to the experiment made the rats familiar to the experimenter followed by 2 days during which the animals were habituated to the hole-board (free exploration of the maze for 15 min each day with access to food pellets). Controlled food restriction was conducted to reduce the weight of rats to 85% of the initial body weight. Tap water was given *ad libitum*. The training was conducted over 3 days (five trials on day 1, four trials on day 2 and a retention trial at day 3) with an intertrial interval of 20 min for individual rats. Trials lasted over 120 s or until all four pellets were found. Possible odor cues of individual rats were removed by cleaning the apparatus with 0.1% Incidin^®^ between trials. Performance of the rats was recorded by a video camera and stored on a computer. The hole visits and removals of pellets were counted for each trial.

Reference memory errors were noted as the number of visits to the unbaited holes. The Reference Memory Index (RMI) was calculated using the formula (first + revisits of baited holes)/total visits of all holes. All behavioral training/testing was performed during the light phase of the light-dark cycle.

Aged rats analyzed in the present study were randomly chosen from a large cohort of 160 male Sprague–Dawley rats 22–24 months of age. Young and old rats were kept as given in a previous publication and were on a calorie restriction diet [R/M Ered II ssniff^®^, Soest, Germany; 5 weeks for young and 4 months for aged animals after they had been fed standard rat chow (R/M-H ssniff^®^, Soest, Germany)] before memory testing. The body weights on the day of sacrifice are shown in Supplementary Table S2. Animals had to visit at least 40 holes during all trials, moreover, young and AU had to find all four baited holes to be accepted for further analysis. AI were defined having mean RMIs derived from trial 6 and 10 lower than one standard deviation (SD) from the mean and AU when having indices one SD higher than the mean. Young rats were analyzed without any grouping.

Data were analyzed with two-way analysis of variance (ANOVA) followed by Tukey’s *post hoc* test (GraphPad Software, San Diego, CA, USA).

### Microscopic Dissection of Dentate Gyrus

Rats were sacrificed after the hole-board test, brains were rapidly removed, hippocampi were placed on a para cooler (RWW Medizintechnik, Germany) at 6°C. Coronal slices were prepared along the longitudinal axis of the hippocampus by a scalpel. Dentate gyrus (DG) was isolated under a stereoscope as described previously (Newton et al., 2005). Tissues were finally transferred into precooled cryogenic tubes and stored at −80°C until tissue was ready for processing.

### Protein Sample Preparation

All homogenization and centrifugation steps were carried out on ice and at 4°C. Brain tissues were homogenized in an ice-cold homogenization buffer [10 mM HEPES, pH 7.5, 300 mM sucrose, 1× Protease Inhibitor Cocktail (PIC, Roche Molecular Biochemicals)] using a Dounce homogenizer; the homogenate was centrifuged at 1,000× *g* for 10 min to remove cell debris and nuclei and the supernatant was collected. The pellet was resuspended again in homogenization buffer and centrifuged at 1,000× *g* for 10 min. The pooled supernatants were then centrifuged at 15,000× *g* for 30 min to obtain the total membrane fraction enriched in synaptosomes and mitochondria. The resulting pellets were washed with 10 mM HEPES, pH 7.5, PIC and solubilized in 50 mM TEAB buffer (Sigma-Aldrich), 7 M urea, 2 M thiourea, 4% CHAPS, 100 mM DTT and PIC. The protein concentration was determined by the Pierce^TM^ 660 nm Protein Assay (Thermo Scientific).

### Tandem Mass Tag (TMT) Based Quantitative Proteomics

Protein samples were digested 18 h with trypsin (Promega) using filter-aided sample preparation (FASP; Wiśniewski et al., 2009) with 75 μg of protein per one reaction. Tryptic peptides were desalted using reversed-phase C18 stage tips (Rappsilber et al., 2007) and reconstituted in 32 μL of 100 mM TEAB (Sigma-Aldrich). The actual amount of peptides was determined by Pierce^TM^ Quantitative Fluorometric Peptide Assay (Thermo Scientific) and 30 μg of peptides were subsequently differentially labeled by TMT10plex^TM^ Isobaric Label Reagent Set (Thermo Scientific) according to the manufacturer’s instructions. The pooled-labeled sample was separated using the Pierce^TM^ High pH Reversed-Phase Peptide Fractionation Kit (Thermo Scientific) into eight fractions and each fraction was reconstituted in 50 μL of 5% formic acid. The peptide fractions were analyzed by liquid chromatography-tandem mass spectrometry (LC-MS/MS) as described previously (Smidak et al., 2017) with two technical replicates per sample and 5 μL injection volume. MS raw data were searched against UniProtKB *Rattus norvegicus* complete proteome database (UP000002494, 31571 sequences, downloaded on February 11th, 2018) using MASCOT 2.4 (MatrixScience) through Proteome Discoverer 2.1 platform (Thermo Scientific) as previously published (Smidak et al., 2017). Raw protein intensities were normalized based on total peptide intensities in the individual TMT channels and scaled according to a TMT 126 reporter control channel to compare all samples across different TMT sets. Significance of changes across groups was tested on individual protein levels by ANOVA (*p* < 0.05) and two-sided *t*-test (*p* < 0.05) to evaluate differences between the corresponding groups using the Perseus statistical package (version 1.5.2.6). Additional filtering was applied based on fold-change with the thresholds [log2 (AU/young protein ratio)] > |0.125| and [log2 (AU/AI protein ratio)] > |0.1| derived from SDs of the fold change values of significant proteins (*p* < 0.05).

### Gene Ontology Analysis

Gene ontology (GO) analysis was performed using the ClueGO Cytoscape plug-in (Bindea et al., 2009). Lists of significantly changed proteins between the groups were used to query KEGG, Reactome and GO—biological function database. ClueGO parameters were set as indicated: Go Term Fusion selected; only display pathways with *p*-values ≤ 0.05; GO tree interval 3–10 levels; GO term minimum # genes, 3; threshold of 4% of genes per pathway. The statistical test used for the enrichment was based on right-sided hypergeometric test with a Benjamini-Hochberg correction and kappa score of 0.6.

### Immunoblot Analysis

Protein samples were separated by electrophoresis on 12% SDS-PAGE gels and transferred to PVDF membranes (GE Healthcare). The western blot procedure was performed as described previously (Shanmugasundaram et al., 2017) with minor modifications. Briefly, membranes were blocked for 1 h in 5% non-fat milk, 1× TBS, 0.1% Tween-20 and probed with the corresponding primary and secondary antibodies. For detection of PRDX6 protein the membrane was incubated in 5% non-fat milk, 1× TBS, 0.1% Tween-20 with 1:1,000 polyclonal rabbit anti-PRDX6 antibody (ab59543, Abcam) 1.5 h at room temperature followed by incubation for 1 h at room temperature with 1:15,000 hP-conjugated anti-rabbit secondary antibody (ab6721, Abcam). Beta-actin as a loading control was detected by incubation in 5% non-fat dry milk, 1× TBS, 0.1% Tween-20 with 1:10,000 anti-β-Actin antibody (4967S, Cell Signalling) 1.5 h at room temperature followed by incubation for 1 h at room temperature in 1:20,000 anti-rabbit polyclonal antibody (ab6721, Abcam). The signals were quantified using ImageJ 1.52a (NIH) software based on the calculation of peak area and the intensity values for PRDX6 bands were normalized across the samples by the actin control. Statistical significance of differences between the group means was evaluated by ANOVA with Tukey correction for multiple comparisons in Prism 6 (GraphPad Software).

## Results

### Behavioral Testing

The effect of aging on spatial memory in 22–24-months-old Sprague–Dawley rats was evaluated using a hole-board spatial memory test. Aged rats were characterized as either impaired (AI) or unimpaired (AU) performers based on their mean RMI derived from trial 6 and 10 in retrieval phases (Figure 1B). We compared learning curves of aged impaired (AI), aged unimpaired and young adults (6 months old). Figure 1C presents the behavioral data in terms of reference memory indices. A two-way ANOVA for repeated measures on RMI revealed a significant overall difference between three groups (*F*_(2,24)_ = 170.8, *p*-value < 0.0001), a significant trial effect (*F*_(9,216)_ = 10.64, *p*-value < 0.0001) and a significant trial × group interaction (*F*_(18,216)_ = 3.373, *p*-value < 0.0001). Tukey *post hoc* comparisons revealed significantly higher RMI of aged unimpaired rats as compared to AI rats (*p*-value < 0.0001), and young compared to AI rats (*p*-value < 0.0001). Trial-by-trial analysis revealed that RMI were significantly higher on day 1 in trial 3 (*p*-value < 0.0006) in AU compared toyoung rats.

### Relative Protein Quantification Using TMT Labeling

To identify proteomic differences in DG between groups, protein samples obtained from behaviorally characterized animals were analyzed using a TMT-based quantitative proteomic approach. The proteomic experiment was performed with nine biological replicates per group distributed between three TMT10plex sets with one, TMT 126 reporter ion, a reference channel in each set was reserved to directly compare the protein intensities across the entire study. Peptides were analyzed in two parallel MS runs representing two technical replicates per biological sample. A total number of 6,513 protein entries was identified and quantified. A number of 471 proteins showed significantly changed levels (*p* < 0.05) between the groups. The comparison between aged and young animals revealed 292 significantly changed proteins between AU vs. young rats, and 310 significantly changed proteins [*p* < 0.05, log2 (protein ratio) > |0.125|] between AI vs. young animals. Of these, 172 proteins showed consistently altered protein levels in old animals regardless of cognitive status, 132 proteins were up-regulated and 40 proteins down-regulated. One-hundred and thirty-eight proteins were specifically changed in AI and 120 proteins in the AU group compared to young ones, suggesting that two aged groups may undergo distinct proteomic changes during aging.

The proteomic differences between AU vs. AI were represented by 77 significantly changed proteins [*p* < 0.05, log2 (protein ratio) > |0.1|]. A number of 20 proteins were significantly different between AU and AI and accordingly were specifically altered in AI compared to young. A set of 13 proteins showed significantly different protein levels between AU and AI and were specifically altered in AU compared to young. Four proteins were significantly different in all three groups. This set of 37 proteins represents proteins relevant to aging and cognitive performance.

**Figure 2:**
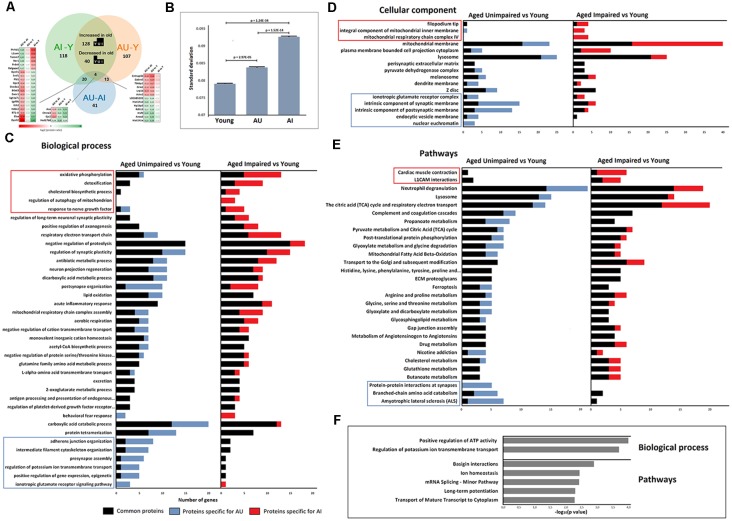
**(A)** Venn diagram showing the relationship between AI vs. young, AU vs. young, and AU vs. AI significant proteins. **(B)** Analysis of variance (ANOVA) of protein levels revealed a significant increase in SD with age and with cognitive impairment (one-way ANOVA, *t*-test *post hoc* test). **(C–E)** Functional enrichment analysis was performed on the list of significantly changed proteins between young and two aging groups using ClueGO-Cytoscape platform (Benjamini–Hochberg *P*-values < 0.05). Displayed gene ontology (GO) terms are the most significant cases from significantly enriched clusters. GO terms in the frames were significantly enriched only in one group AU vs. young or AI vs. young. **(F)** Significantly enriched GO terms in the set of 77 proteins with different protein levels between AU and AI.

All identified proteins and significantly changed proteins between the animal groups are included as Supplementary Information (Supplementary Table S1), and the overlap between the groups is represented in the proportional Venn diagram in Figure 2A.

### Identification of Aging Markers

The sensitivity of our approach allowed detection of 30 previously established age markers within significantly changed proteins between young and aged [JenAge Ageing Factor Database (Huhne et al., 2014)]. For example, we detected aging-dependent upregulation of the enzyme beta-galactosidase (Ori et al., 2015), Apod (Sanchez et al., 2015), Complement C1q subunits a and c (Stephan et al., 2013), Gstp1 (Martínez-Lara et al., 2003), GFAP (Bernal and Peterson, 2011), a widely used marker of brain aging which demonstrates that our method recapitulates previous findings and can detect biologically meaningful changes.

### Functional Annotation of Proteins Altered During Aging

In an attempt to translate proteomic changes in aging into functional consequences, functional enrichment analysis of differentially expressed proteins using the ClueGo Cytoscape plug-in was performed (Bindea et al., 2009).

GO analysis confirmed that different functional clusters are affected in DG of aged AI and AU compared to young rats, but also underlined the existence of common aspects of altered protein functions independent of cognitive status.

Analyses revealed several biological processes that have been previously associated with age and appeared enriched in both aged groups. This includes energy metabolism related processes, lipid oxidation, negative regulation of proteolysis, regulation of synaptic plasticity and acute inflammatory response (Figure 2C). Enrichment analysis for cellular component revealed pronounced changes in the expression of mitochondrial and lysosomal proteins (Figure 2D).

As shown in Figure 2E, common enriched biological pathways were proposed to be mainly associated with the immune system, lysosomes and energy metabolism. Furthermore, some pathways such as the TCA cycle and respiratory electron transport, ECM proteoglycans, and Mitochondrial Fatty Acid Beta-Oxidation, which have been widely reported to be associated with the aging process, were also significantly enriched.

Lysosomes and lysosomal proteases have been shown to change their properties in the aging brain, moreover, lysosomal dysfunction is associated with many age-related diseases like Parkinson’s and Alzheimer’s disease. In line with previous studies, GFAP, cathepsins, Pon2, Glb1 protein levels were consistently increased in aged animals (Nakanishi et al., 1997; Bernal and Peterson, 2011; Ori et al., 2015).

Enrichment analysis revealed also specific functional clusters enriched only in one protein set. Biological processes specifically enriched in AU during aging are related to cytoskeleton organization, adherens junction, synaptic function, potassium ion transport, glutamate receptor signaling pathway and epigenetic processes that positively regulate gene expression. Biological processes specifically enriched in AI during aging were oxidative phosphorylation, cholesterol biosynthesis, regulation of mitophagy, response to nerve growth factor and detoxification.

Enrichment analysis on 77 proteins with significantly different protein levels between AU and AI groups for biological processes indicated two clusters: positive regulation of ATP activity and regulation of potassium ion transmembrane transport. Pathway analysis revealed enrichment of pathways related to long-term potentiation, basigin interactions, mRNA transport, splicing and ion homeostasis (Figure 2F).

Several GO terms related to mitochondrial function were enriched in aging. Mitochondrial dysfunction and mitochondria-derived ROS has been considered a major contributor to aging and age-related disorders including neurodegenerative disorders. Twenty percent of all proteins significantly changed in old animals compared to young ones were annotated using MitoMiner (Smith and Robinson, 2016), a database of the mitochondrial proteome, as experimentally proven mitochondrial elements. All of the subunits of the Electron Transfer Chain that exhibited changes in protein levels during aging were up-regulated, which is in line with previous findings (Stauch et al., 2014). However, dysregulation of the oxidative phosphorylation process seems to be at a lowerextent in AU.

**Figure 3:**
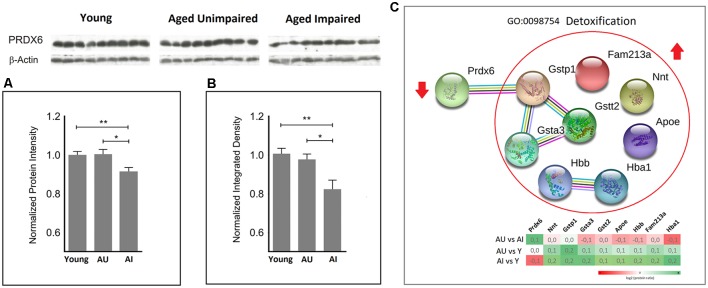
Quantitative proteomic analysis **(A)** revealed significantly increased protein levels of peroxiredoxin 6 (PRDX6) in the AU vs. AI and young rats vs. AI (two-sided *t*-test). This finding was subsequently confirmed by immunoblot analysis **(B)**. A normalized protein ratio is represented by group means of normalized protein intensities vs. young control and is expressed as mean ratio ± SEM (*p-value < 0.05; **p-value < 0.01; *n* = 9). **(C)** Representative GO term/cluster highlighted in enrichment analysis. Associated proteins are presented as association network using STRING 11. PRDX6 was the only protein in this cluster with decreased protein levels in AI compared to young and AU rats.

Since mitochondria are the major source of cellular ROS and markedly contribute to age-associated damage, increased ROS production during aging may elicit a compensatory protective response. Enrichment analysis highlighted detoxification processes, among other protein functional categories as discriminative between AU and AI. This cluster contains also glutathione S-transferases (Gsta3, Gstp1, Gstt2) representing a primary line of cellular defense against toxicities of electrophiles and ROS/RNS (Tew and Townsend, 2012; Figure 3C). A closer examination of the direction of protein changes revealed up-regulation of all proteins in this cluster in aged animals, except PRDX6, a major enzyme involved in the detoxification of ROS, which was down-regulated in AI compared to AU and young rats (Figure 3).

### PRDX6 Discriminates AU From AI

PRDX6 was unambiguously identified with 15 unique peptides and 81% sequence coverage. PRDX6 showed significantly increased protein levels in the AU as compared to AI with log2 (Fold Change) value 0.136 and *p* = 0.019, and young rats vs. AI with log2 value 0.133 and *p* = 0.009 (Figure 3A). This finding was confirmed by subsequent western blotting analysis (*p*-value = 0.013 for AU vs. AI and 0.004 for young vs. AI; Figure 3B, Supplementary Figure S1).

### Variance in Protein Levels Is a Discriminating Factor Between AI and AU

Recent studies showed that stochastic deregulation of gene expression increases during aging (Végh et al., 2014). To test whether stochasticity in protein levels is a discriminative factor for cognitive impairment during aging, we measured variance in protein levels in all three groups. We calculated the SD of the average protein intensities per protein and indeed observed a significant increase in SD with age and with cognitive impairment (one-way ANOVA test, *p*-value = 0; *t*-test *post hoc* test showed significant changes between all three groups; Figure 2B). Total peptide intensities did not change with age and cognitive impairment (one-way ANOVA test, *p*-value = 0.978), which excluded variation in total protein levels as a reason for the observed SD values.

## Discussion

Cognitive decline is often viewed as a natural part of the aging process in humans and animals, however, there is a notable heterogeneity in cognitive abilities in “healthy” aging (Gallagher and Rapp, 1997; Gallagher et al., 2006; Gerstein et al., 2013). Aged Sprague-Dawley rats exhibit individual differences in cognitive functions and can be subdivided into impaired and unimpaired in spatial memory tasks.

In the present study, a large set of aged Sprague–Dawley rats was tested in a hole-board test. Due to the aging fragility only one paradigm could be used in order to avoid excess stress. A spatial hole-board task can be used to assess cognitive abilities such as spatial discrimination, memory and learning. Lasting reference memory formed by learning on the hole-board has been related to hippocampal long-term potentiation and it has been shown in several studies that the DG is specifically involved (Uzakov et al., 2005; Korz and Frey, 2007; Woldeit and Korz, 2010). Animals that fulfilled our criteria (mean RMIs from trial 6 and trial 10 ± 1 SD) were characterized as either AI or AU. The impaired group showed decline in memory, however, the unimpaired group performed on par with the spatial memory performance of the young animals. According to unpublished own results, most of AU are superior to the rest of the population already at 6 months of age. This may partially explain better performance of AU compared to young animals on day 1, however, there was no difference between AU and young animals on day 2 and during retrieval on day 3. It is important to notice that a large series of studies in aging neurobiology simply compare young to old animals ignoring that the “aging” panel *per se* is a heterogeneous group, or subdivide a small group of old animals without any intersection. According to own results, only about 10% of the aged rat population assigns to either impaired or unimpaired.

It has been repeatedly demonstrated—either at the transcriptomic or proteomic level—that among brain regions relevant for spatial memory, aging mostly affects the hippocampus, in particular, the DG (Ianov et al., 2017; Hamezah et al., 2018). Ianov et al. (2017) have shown that out of three hippocampal regions the DG exhibits most robust transcriptional changes during aging. Therefore, we selected the DG for quantitative proteomic analysis. A TMT labeling-based approach allowed identification and quantification of a total of 6,514 protein entries. Four-hundred and thirty proteins showed altered protein levels at least in one aging group, of this 172 proteins were consistently changed in both groups, 138 and 120 proteins were specifically changed in AI and AU, respectively. In order to understand the potential impact of the protein changes common and specific for aging groups, proteins altered during aging in AU and AI animals were analyzed for biological process and cellular component ontologies, and pathways using ClueGO. Our results demonstrate that not all changes underline chronological aging, but there are changes in protein levels specifically coupled to cognitive status of aged rats.

The current enrichment analysis highlighted significant alteration in levels of mitochondrial proteins involved in energy metabolism during brain aging. Mitochondrial function and energy metabolism has long been recognized to deteriorate during aging (Pulliam et al., 2013; Payne and Chinnery, 2015). The synapses rely on high ATP levels for maintenance of membrane potential and organization of synaptic vesicles (Murthy and De Camilli, 2003). In addition to the role for ATP production, mitochondria are involved in maintenance of Ca^2+^ homeostasis in the synapse, critical for the regulation of neurotransmission (Sheng and Cai, 2012). Stauch et al. (2014) reported that despite proteomic alterations in synaptic mitochondria of old mice and increased substrate utilization by complex II and complex IV, they maintained overall mitochondrial functions and changes in the synaptic mitochondrial proteome are likely adaptive. However, consistent with our study, they detected increased levels of antioxidant enzymes indicating a response to increased ROS in the aged animals. Mitochondrial ROS and RNS production have been reported extensively in the literature and the electron transport chain (ETC) has been conceded as one of the main intracellular sources of superoxide anions (Boveris, 1977; Radi et al., 2002; Gutierrez et al., 2006). Under physiological conditions, ROS are signaling molecules involved in processes such as immune response, inflammation, as well as synaptic plasticity and hence, in normal cognitive function (Massaad and Klann, 2011). ROS have been shown to regulate synaptic plasticity-related signaling molecules, receptors, transporters and channels (Angelova and Müller, 2006; Huddleston et al., 2008; Shetty et al., 2008; Hota et al., 2010). However, when an imbalance between ROS production and detoxification occurs, ROS production may override the antioxidant defense leading to impaired cellular functions. Consequences of redox imbalance are mitochondrial dysfunction, lipid oxidation, pro-inflammatory activity, increased apoptosis, and dampened synaptic plasticity. In the present study biological processes associated with lipid oxidation, inflammation and regulation of synaptic plasticity were enriched as common to both aged groups.

Probably as a response to increased levels of ROS and toxic compounds derived from peroxidation reactions, a detoxification cluster, which contains proteins with antioxidant activity was specifically enriched in AI. PRDX6, a member of this cluster, showed significantly decreased protein levels in a synaptosomal enriched fraction from DG in the AI vs. AU and young. The role of PRDX6 in oxidative stress was described above in the introduction section. Decline of PRDX6 during aging has been associated with ocular pathobiology induced by age-related oxidative stress (Kubo et al., 2017; Chhunchha et al., 2018). Chhunchha et al. (2017) proposed PRDX6 as a potential therapeutic target to protect the trabecular meshwork from age-dependent oxidative stress and accumulation of ROS by balancing redox-homeostasis to prevent ocular disorders.

In addition to its known antioxidant activity, PRDX6 has been identified as a regulator of mitophagy (Ma et al., 2016). Ma et al. (2016) showed that a loss of mitochondrial potential promotes recruitment of PRDX6 to mitochondrial membrane, stabilization of Pink1 with subsequent induction of mitophagy through the Pink1-Parkin pathway. Previous studies have shown that mitophagy, a selective macroautophagic process for removal of damaged mitochondria, is impaired during aging (Palikaras and Tavernarakis, 2012; Fivenson et al., 2017). This is supported by evidence that genetically increased autophagy delays aging (Schiavi et al., 2015; Diot et al., 2016).

Regulation of mitochondrial autophagy was one of the biological processes specifically altered during aging in AI. A GO cluster was represented by Tomm7 with significantly decreased protein levels in AI compared to AU and young. Tomm7 is a known regulator of mitophagy through interaction with Pink1. Based on current results, dysregulation of regulators of mitophagy may take place during cognitive aging. Moreover, alteration of lysosomal proteins and regulation of proteolysis were also common features for aged animals. Integrity of the autophagosomal-lysosomal network appears to be critical in the progression of aging (Rajawat et al., 2009).

Aging is associated with increased heterogeneity of gene expression (Somel et al., 2006; Végh et al., 2014) and herein, analysis of variation of protein levels could distinguish between AI and AU. Both aged groups showed increased variation of protein levels, however, there was a significant increase in variation of protein levels in AI compared to young and AU (Figure 2B). This represents the first evidence that heterogeneity not only increases with aging but is also a discriminative factor for cognitive impairment in aging.

Taken together, in the hole-board test, a non-invasive spatial memory task, aged individuals with unimpaired in contrast to impaired memory showed PRDX6 in the DG comparable to those of young individuals. This points to a role of this antioxidant enzyme for keeping memory functions during the aging process. We have shown this finding by unambiguous PRDX6 identification and quantification by a proteomics LC-MS method in a synaptosomal-enriched fraction and this observation was confirmed by immunoblotting.

The current study not only forms the basis for the design of future studies and interpretation of previous work but also challenges further work on this antioxidant enzyme in aging neuroscience and neuropharmacology. There is already a legion of publications on medications acting on the antioxidant defense claiming to improve learning and memory, herein we provide a potential further well-defined target for the antioxidant pharmacology.

## Data Availability

The mass spectrometry proteomics data have been deposited to the ProteomeXchange Consortium *via* the PRIDE (Perez-Riverol et al., 2019) partner repository with the dataset identifier PXD013172.

## Ethics Statement

The study was carried out in accordance with the recommendation of the European Communities Council Directive of 24 November 1986 (86/609/EEC) evaluated by the ethics committee of the Medical University of Vienna, Austria. The protocol was approved by Federal Ministry of Education, Science and Culture, Austria.

## Author Contributions

JL has written the manuscript and analyzed proteomic data. RS carried out analysis on LC-MS and the immunoblotting. JM and DF carried out animal experiments. VK supervised animal experiments and handled data from behavioral analysis. HH bred the animals, has taken care of veterinary issues and co-supervised the behavioral analysis. GL initiated and designed the entire research program on aging of the rat and participated in writing the manuscript.

## Conflict of Interest Statement

The authors declare that the research was conducted in the absence of any commercial or financial relationships that could be construed as a potential conflict of interest.

## Supplementary Material

FIGURE S1 Original Western blot used for Figure 3B.

TABLE S1 The list of proteins identified in the quantitative proteomic study and the proteins with significantly changed levels between young and aged unimpaired and aged impaired rats.

TABLE S2 Weight of the rats on the day of sacrifice.
