# Supplementary figures and images for: Dentate Gyrus Peroxiredoxin 6 Levels Discriminate Aged Unimpaired From Impaired Rats in a Spatial Memory Task
Source: Front Aging Neurosci. 2019 Jul 31;11:198. doi: 10.3389/fnagi.2019.00198 (PMC6684764; doi:10.3389/fnagi.2019.00198)

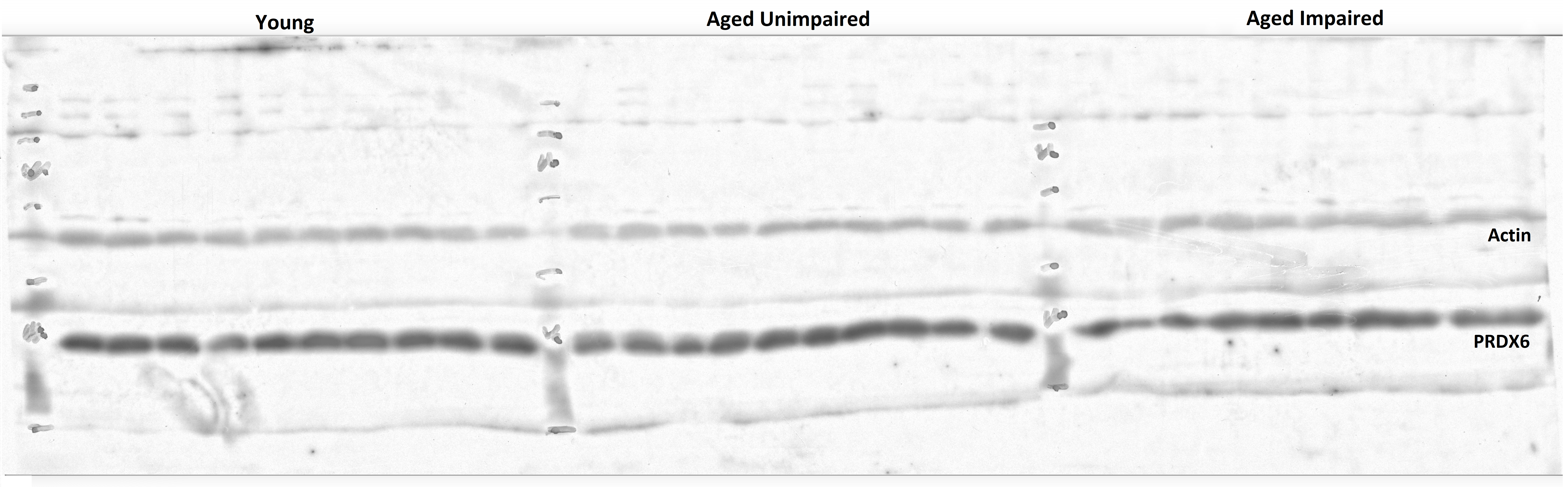

Supplement: FIGURE S1 — Original Western blot used for Figure 3B. [file Image_1.TIF]
